# Supplementary material for: Students’ preparedness for disasters in schools: a systematic review protocol
Source: BMJ Paediatr Open. 2020 Dec 4;4(1):e000913. doi: 10.1136/bmjpo-2020-000913 (PMC7722369; doi:10.1136/bmjpo-2020-000913)
Supplement: Supplementary data [file bmjpo-2020-000913supp002.pdf]

Additional file: Draft of the search strategy utilized in each selected database.

Search Strategy	
**Population**	child *OR* adolescent *OR* teen *OR* student *OR* boy *OR* girl *OR* kid *OR* pediatric
AND	
**Interest**	floods *OR* hurricanes *OR* tornadoes *OR* volcanoes *OR* earthquakes *OR* tsunamis *OR* storms *OR* hazards *OR* risks *OR* fire *OR* bushfire *OR* landslide *OR* sandstorm *OR* drought *OR* snowstorm *OR* avalanche *OR* thunderstorms *OR* COVID-19 *OR* Influenza *OR* Zika *OR* malaria *OR* Ebola *OR* epidemic *OR* famine *OR* mass shooting *OR* industrial accident
	AND
	preparedness *OR* education *OR* readiness *OR* drill
AND	
**Context**	school *OR* class

Search Strategy – PubMed (May 5, 2019)

Search:

#1 (((child[Title/Abstract] OR adolescent[Title/Abstract] OR teen[Title/Abstract] OR student[Title/Abstract] OR boy[Title/Abstract] OR girl[Title/Abstract] OR kid[Title/Abstract] OR pediatric[Title/Abstract])

AND

#2 (floods[Title/Abstract] OR hurricanes[Title/Abstract] OR tornadoes[Title/Abstract] OR volcanoes[Title/Abstract] OR earthquakes[Title/Abstract] OR tsunamis[Title/Abstract] OR storms[Title/Abstract] OR hazards[Title/Abstract] OR risks[Title/Abstract] OR fire[Title/Abstract] OR bushfire[Title/Abstract] OR landslide[Title/Abstract] OR sandstorm[Title/Abstract] OR drought[Title/Abstract] OR snowstorm[Title/Abstract] OR avalanche[Title/Abstract] OR thunderstorms[Title/Abstract] OR COVID-19[Title/Abstract] OR Influenza[Title/Abstract] OR Zika[Title/Abstract] OR malaria[Title/Abstract] OR Ebola[Title/Abstract] OR epidemic[Title/Abstract] OR conflicts[Title/Abstract] famine[Title/Abstract] OR mass shooting OR [Title/Abstract] OR industrial accidents [Title/Abstract]))

AND

#3 (preparedness[Title/Abstract] OR education[Title/Abstract] OR readiness[Title/Abstract] OR drill[Title/Abstract]))

AND

#4 (school[Title/Abstract] OR class[Title/Abs act]) (((((((("child"[Title/Abstract] OR "adolescent"[Title/Abstract]) OR "teen"[Title/Abstract]) OR "student"[Title/Abstract]) OR "boy"[Title/Abstract]) OR "girl"[Title/Abstract]) OR "kid"[Title/Abstract]) OR "pediatric"[Title/Abstract])
