# Supplementary material for: Students’ preparedness for disasters in schools: a systematic review protocol
Source: BMJ Paediatr Open. 2020 Dec 4;4(1):e000913. doi: 10.1136/bmjpo-2020-000913 (PMC7722369; doi:10.1136/bmjpo-2020-000913)
Supplement: Author's manuscript [file bmjpo-2020-000913.draft_revisions.pdf]

BMJ Paediatrics Open

BMJ Paediatrics Open is committed to open peer review. As part of this commitment we make the peer review history of every article we publish publicly available.

When an article is published we post the peer reviewers' comments and the authors' responses online. We also post the versions of the paper that were used during peer review. These are the versions that the peer review comments apply to.

The versions of the paper that follow are the versions that were submitted during the peer review process. They are not the versions of record or the final published versions. They should not be cited or distributed as the published version of this manuscript.

BMJ Paediatrics Open is an open access journal and the full, final, typeset and author-corrected version of record of the manuscript is available on our site with no access controls, subscription charges or pay-per-view fees (http://bmjpaedsopen.bmj.com).

If you have any questions on BMJ Paediatrics Open's open peer review process please email info.bmjpo@bmj.com

BMJ Paediatrics Open

Students' Preparedness for Disasters in Schools: A Systematic Review Protocol

Journal:	*BMJ Paediatrics Open*
Manuscript ID	bmjpo-2020-000913
Article Type:	Protocol
Date Submitted by the Author:	13-Oct-2020
Complete List of Authors:	Seddighi, Hamed; University of Social Welfare and Rehabilitation Science<br>Sajjadi, Homeira; University of Social Welfare and Rehabilitation Science<br>Yousefzadeh, Sepideh; RUG, Campus Friesland<br>Lopez, Monica; RUG<br>Vameghi, Meroe; University of Social Welfare and Rehabilitation Science<br>Rafiey, Hassan; University of Social Welfare and Rehabilitation Science<br>Khankeh, Hamid Reza; University of Social Welfare and Rehabilitation Science<br>Garzon Fonseca, Magdalena; University of Groningen,
Keywords:	Adolescent Health

SCHOLARONE™
Manuscripts

I, the Submitting Author has the right to grant and does grant on behalf of all authors of the Work (as defined in the below author licence), an exclusive licence and/or a non-exclusive licence for contributions from authors who are: i) UK Crown employees; ii) where BMJ has agreed a CC-BY licence shall apply, and/or iii) in accordance with the terms applicable for US Federal Government officers or employees acting as part of their official duties; on a worldwide, perpetual, irrevocable, royalty-free basis to BMJ Publishing Group Ltd ("BMJ") its licensees and where the relevant Journal is co-owned by BMJ to the co-owners of the Journal, to publish the Work in this journal and any other BMJ products and to exploit all rights, as set out in our licence.

The Submitting Author accepts and understands that any supply made under these terms is made by BMJ to the Submitting Author unless you are acting as an employee on behalf of your employer or a postgraduate student of an affiliated institution which is paying any applicable article publishing charge ("APC") for Open Access articles. Where the Submitting Author wishes to make the Work available on an Open Access basis (and intends to pay the relevant APC), the terms of reuse of such Open Access shall be governed by a Creative Commons licence – details of these licences and which Creative Commons licence will apply to this Work are set out in our licence referred to above.

Other than as permitted in any relevant BMJ Author's Self Archiving Policies, I confirm this Work has not been accepted for publication elsewhere, is not being considered for publication elsewhere and does not duplicate material already published. I confirm all authors consent to publication of this Work and authorise the granting of this licence.

Confidential: For Review Only

Students' Preparedness for Disasters in Schools: A Systematic Review Protocol

Hamed Seddighi, Homeira Sajjadi, Sepideh Yousefzadeh, Mónica López López, Meroe Vameghi, Hassan Rafiey, Hamidreza Khankeh, Magdalena Garzon Fonseca

Hamed Seddighi: Student Research Committee, University of Social Welfare and Rehabilitation Sciences, Tehran, Iran,Hseddighi@gmail.com

Homeira Sajjadi: Social Determinants of Health Research Center, University of Social Welfare and Rehabilitation Sciences, Tehran, Iran, Safaneh_s@yahoo.com

Sepideh Yousefzadeh: Population Research Centre, Faculty of Spatial Sciences, University of Groningen, Groningen, the Netherlands, s.yousefzadeh@rug.nl

Mónica López López: Faculty of Behavioural and Social Sciences, University of Groningen, Groningen, the Netherlands, m.lopez.lopez@rug.nl

Meroe Vameghi: Social Welfare Management Research Center, University of Social Welfare and Rehabilitation Sciences, Tehran, Iran, m_vameghi@yahoo.com

Hassan Rafiey: Social Welfare Management Research Center, University of Social Welfare and Rehabilitation Sciences, Tehran, Iran, hassan441015@gmail.com

Hamidreza Khankeh: Health in Emergency and Disaster Research Center, University of Social Welfare and Rehabilitation Sciences, Tehran, Iran, hamid.khankeh@ki.se

Magdalena Garzon Fonseca (Corresponding author): Department of Child & Family Welfare, Graduate School of Behavioral Sciences, University of Groningen, m.garzon.fonseca@rug.nl

Keywords: Disasters, Natural hazards, Climate change, Children, Preparedness

Word count: 2200

Confidential: For Review Only

Abstract

Introduction

Children are one of the most vulnerable groups in natural disasters. Improving students' knowledge and skills to prepare for natural disasters can play a major role in children's health. School as a place to teach children can make a significant contribution to provide the necessary skills. This study aims to identify the effects, strengths and weaknesses of interventions in schools to prepare children for natural disasters.

Methods and analysis

We use Preferred Reporting Items for Systematic Review and Meta-Analysis (PRISMA) guidelines to develop a protocol for this systematic review. The included studies will report on the results of interventions targeting “schoolchildren” defined as individuals between 4 and under 18 years old studying in schools. Different electronic databases will be used for a comprehensive literature search, including MEDLINE, Web of Science, CINAHL, PsycINFO, Cochrane Register of Controlled Trials (CENTRAL) and EMBASE to identify the records that match the mentioned inclusion criteria published till December 2020. The main search terms are "natural disasters", "preparedness", "children", and "school". Four types of data will be extracted from the qualified studies including study characteristics (study design, year of publication, and geographical region where the study was conducted), participant characteristics (sample size, age, and gender), intervention characteristics (aim of intervention, intervention facilitators and barriers), and intervention outcomes. The quality appraisal of the selected papers will be conducted using Cochrane Collaboration’s Risk of Bias for quantitative studies and Critical Appraisal Skills Program (CASP) checklist for qualitative studies. We use a narrative synthesis for this systematic review. The narrative synthesis refers to an approach to systematic reviews which focuses mostly on applying words and texts to summarize and explain findings.

Ethics and dissemination

This paper is a part of a Ph.D. thesis at University of Social welfare and Rehabilitation Sciences with ethics code IR.USWR.REC.1399.008 approved by the Ethics Committee of the above-mentioned university.

PROSPERO registration number

Registration number: CRD42020146536

Confidential: For Review Only

What is already known on this topic?

- Although various studies have been conducted on the interventions in schools to prepare them for natural disasters, so far no systematic review has been conducted to identify methods and challenges.
- It is unclear differences of processes and outcomes in term of geographical location, age, gender, diversity, and type of natural disaster.

What this study hopes to add?

- First study that provides a comprehensive summary of studies investigating School-based education programs for preparing children for natural disasters.
- Provide useful information for policy makers, governments, researchers, and humanitarian organizations about state of art of programs for preparing children for natural disasters around the world.
- Highlight closing the gaps for preparing children for natural disasters such as earthquake, flood, and pandemics.

Confidential: For Review Only

1. Introduction

In recent decades, more natural hazards have occurred[1]. Natural hazards are one of the major threats to children's health[2]. According to the origin, natural hazards are categorized as geophysical (earthquakes, landslides, tsunamis and volcanic activity), hydrological (avalanches and floods), climatological (extreme temperatures, drought and wildfires), meteorological (cyclones and storms/wave surges) or biological (disease epidemics and insect/animal plagues)[3]. Children are one of the most vulnerable groups during and after disasters[4]. disasters triggered by natural hazards can seriously damage children's physical and mental health[5]. It also harms children indirectly, so that its outcomes may last a lifetime[6,7]. In addition, disasters destroy children's educational infrastructure and disrupt learning in children [8]. Moreover, following disasters, violence against children increases in sexual, physical, psychological and neglect forms[9]. Child trafficking has been reported as one of the impacts of disasters in the affected areas[10]. Children who have lost their parents or caregivers and the children whose parents cannot afford to live will have to work after disasters[10,11]. Early marriage is another case reported in the affected areas, especially for girls[12,13]. Each of the above-mentioned cases also affects social, economic, and political structures[14]. These consequences will last for generations[15]. Although timely response and good governance have a significant effect in preventing such consequences, children's readiness can be one of the main solutions to reduce post-disaster losses[16,17].

In previous decades, most managers and decision-makers would think that children are not able to take care of themselves and they need help from adults[18]. Therefore, most interventions for disaster preparedness were defined for adults. But now researchers have found that children can respond appropriately to an accident and stay healthy[19]. The United Nations has recognized raising the children's awareness as one of the main pillars of children's readiness[19].

School as a place where almost all children gather to enjoy the opportunity to learn, can be the best place to prepare them for disasters[20]. The required knowledge and skill can be provided to the child in various ways in schools, one of which is practical training and skills development[21]. Although various studies have been conducted on the interventions in schools to prepare the children for natural hazards, no systematic review has been yet conducted to identify methods and challenges.

1.1. Aim of Review

The main purpose of this study is to identify the effects, facilitators and barriers of educational programs in schools to prepare children for natural hazards. In addition, the differences of processes and outcomes in terms of geographical location, age, gender, diversity, and type of natural disaster are investigated.

2. Method

We use Preferred Reporting Items for Systematic Review and Meta-Analysis (PRISMA) guidelines to develop the protocol for this systematic review (Supplementary Material 1) [22] registered in PROSPERO International Prospective Register of Systematic Reviews (registration number: CRD42020146536). In addition, Cochrane guidance is applied in the development of a systematic review protocol to specify the research questions using PICO-elements (Population, Interventions, Comparators, and Outcomes). Studies will be assessed against clearly defined

Confidential: For Review Only

criteria to determine their inclusion or exclusion in the review. Finally, the findings of the included studies will be assessed and reported[23].

2.1. Eligibility

2.1.1. Inclusion Criteria

a) Type of participants:

The included studies will report on the results of interventions targeting “schoolchildren” defined as individuals under 18 years old studying in schools.

b) Context/Setting:

The age of compulsory school entry is various in the world but it is normally 4 to 7 years old. For this review we will consider the children aged 4 to 18 in public or private schools.

c) Type of Interventions:

The included studies will report on the interventions delivered in schools with a focus on natural disaster preparedness education. Various interventions with different duration, timing, and modality will be acceptable. Acceptable interventions are those delivered in full or in part in the schools. Such interventions were delivered by teachers or other people playing a natural disaster preparedness role. Schools are chosen as the primary setting because many programs for children are delivered in schools (i.e. shakeout drills).

d) Type of studies:

The studies included in this review will be as the following: qualitative studies (those evaluating the process of school-based educational interventions for preparedness against natural hazards), observational studies (including case-control, cohort and cross-sectional studies) and clinical trials (including non-randomized and randomized controlled trials) examining the effect of disaster preparedness education interventions on school students.

e) Types of publication:

The study will include empirical research works published in English language peer-reviewed journals or conference proceedings that are accompanied with full-length peer-reviewed papers.

f) Outcome measures:

The included studies will report on the interventions the primary outcome of which is an appropriate focus on the children's preparedness (knowledge, attitude, and behavior) for natural hazards.

The primary goal of all included interventions is to improve children preparedness for natural hazards. For this reason, included studies shall report on at least one educational instrument applied to measure children’s preparedness.

2.1.2. Exclusion Criteria

a) Type of participants:

The study will exclude individuals under 4 and above 18 years old.

b) Context/setting:

Studies will be excluded if they report interventions delivered in settings other than the schools.

c) Type of interventions:

Studies will be excluded if (i) they report on human-made disasters (i.e. nuclear accidents, mass shooting, industrial accident and the like) and/or (ii) they do not include a natural hazards’ preparedness intervention for improving skills and changing knowledge, attitude, and behavior,

Confidential: For Review Only

(iii) they report on the interventions delivered without a school-based component (i.e. natural hazards' preparedness education for children in museum)

d) Type of studies:

Studies will be excluded if they are not original intervention studies, they are published in journals that are not peer-reviewed, or they are not published in the English language. They will be excluded if they do not report on a natural hazards' preparedness. Studies that report conference abstracts (without a corresponding full-length peer-reviewed paper) and unpublished research (e.g., unpublished dissertations/theses) will not be included.

e) Outcome measures:

There are two criteria for exclusion including (i) report on interventions about human-made disasters, (ii) the works which do not include relevant outcome measures, (iii) when the intervention results and outcomes do not appear to have an appropriate effect on preparedness.

2.2. Search Strategy

We will use electronic databases for comprehensive literature search including MEDLINE, Web of Science, CINAHL, PsycINFO, Cochrane Register of Controlled Trials (CENTRAL) and EMBASE to identify the records meeting the mentioned inclusion criteria and published till April 2020. Different keywords for the systematic search were identified during the initial literature search. The main search terms are "natural hazards", "preparedness", "children", and "school". The combinations of keywords related to population, interest, and context and the draft of MEDLINE search strategy is presented in appendix 1. Search terms will be combined with the appropriate Boolean operators and the search will be based on titles, abstracts, and keywords.

2.3. Selection Processes

Two reviewers will participate in the selection process. An Endnote desktop will be used to store references and subsequently identify and remove duplicates. For finding eligible studies, abstracts and full texts will be reviewed. Two reviewers will then separately scan abstracts and full texts of currently eligible studies against the eligibility requirements of the research, taking into consideration the type of intervention, sample population and the recorded outcomes. The two reviewers must come to an agreement through discussion and if they fail to reach a compromise, a third reviewer may have a resolution.

2.4. Data Extraction

Two reviewers shall individually perform data retrieval, and agreement will be achieved through conversation. The following data will be extracted from the qualified studies:

- Study characteristics: study design, year of publication, and geographical location of study conduct
- Participant characteristics: sample size, age (e.g., mean with standard deviation, range), and gender
- Intervention characteristics: aim of intervention, intervention facilitators and barriers,
- Intervention outcome

2.5. Quality Appraisal

The quality appraisal of the selected papers will be conducted separately by two reviewers using Cochrane Collaboration's Risk of Bias (CCRB)[24,25]. CCRB will be utilized for measuring risk of bias for selected articles with randomized design. Bias is surveyed in five dimensions, including selection bias, performance bias, detection bias, attrition bias, and reporting bias.

For assessing risk of bias in qualitative studies, we will use the Critical Appraisal Skills Program (CASP) checklist recommended by the Cochrane Collaboration for qualitative literature[26]. This tool has 10 questions (9 questions addressing quality and 1 addressing "value").

2.6. Data Synthesis

A narrative synthesis will be used for this systematic review. The narrative synthesis refers to an approach to systematic reviews that focuses mostly on use of words and texts to summarize and

Confidential: For Review Only

explain findings[27]. It is usually considered as the "second best" approach for synthesis in systematic reviews. In fact, this approach is a significant method to interpret findings extensively used in policy and practice. This approach will discuss on the effects of interventions and factors shaping the implementation of interventions. According to Popay et al. (2006), in order to conduct the narrative synthesis four steps shall be followed [27]:

- Developing a theory of how the intervention works, why and for whom,
- Developing a preliminary synthesis of findings of included studies,
- Exploring relationships in the data,
- Assessing the robustness of the synthesis.

the first step will contribute to the interpretation of the study findings and to assess how widely the findings may be applicable. The second step is developing a preliminary synthesis. In this step the aim is to identify and list facilitators and barriers to implementation of interventions on children's preparedness in schools for natural hazards. Exploring relationships in the data will be the third step. This step aims to explain the facilitators and/or barriers to successful implementation across the included studies and to understand how and why the interventions have an effect. In the final step, the robustness of the synthesis product will be assessed. The aim of this step is to provide an assessment of the strength of the evidence in order to draw conclusions about the facilitators and/or barriers to implementation identified in the synthesis.

for developing a preliminary synthesis, three methods will be used including textual descriptions, grouping and clusters, and tabulation.

In textual descriptions, a descriptive paragraph on the included studies will be presented. In grouping method, we aim to cluster findings to aid the process of analyzing and finding patterns. Studies will be categorized according to the type of interventions, contexts, and outcomes. In addition, tabulation will be used to summarize some findings.

3. Conclusions

In the planned systematic review, we aim to identify outcomes, facilitators, and barriers of school-based education programs for preparing children for natural hazards. Such a review can raise decision-makers' knowledge of disaster preparedness for children. Also, it can be effective in identifying best practices and the challenges of intervention through the world. Recognizing best practices will be an opportunity for countries researching for interventions. Researchers can also find solutions to remove the barriers to preparedness of children for natural hazards.

Confidential: For Review Only

Supplementary Materials
PRISMA checklist
Search strategy

Contributors
All authors reviewed, contributed to and approved the final version of the manuscript. All authors have read and agreed to the published version of the manuscript.

Funding and Ethics
This paper is a part of a Ph.D. thesis at University of Social welfare and Rehabilitation Sciences with ethics code IR.USWR.REC.1399.008 approved by the Ethics Committee of the mentioned university.

Competing interests: Not applicable.

Patient and public involvement: Not applicable.

Patient consent for publication: Not applicable.

Confidential: For Review Only

References

1. Joshi N, Roberts R, Tryggvason A. Are fatality potentials of natural disasters increasing? Examining how natural and human factors affect societal vulnerability. Paper presented at: Geophysical Research Abstracts2019.
2. Seddighi H, Sajjadi H, Salmani I. Child-Friendly Humanitarian Logistics in Natural Disasters: A Letter to the Editor. *Iranian Red Crescent Medical Journal.* 2019;21(8).
3. Guha-Sapir D, Vos F, Below R, Ponserre S. Annual disaster statistical review 2010. *Centre for Research on the Epidemiology of Disasters.* 2011.
4. Benevolenza MA, DeRigne L. The impact of climate change and natural disasters on vulnerable populations: A systematic review of literature. *Journal of Human Behavior in the Social Environment.* 2019;29(2):266-281.
5. Subedi S, Bartels S, Davison C. Emotional and physical child abuse in the context of natural disasters: a focus on Haiti. *Disaster medicine and public health preparedness.* 2019;13(5-6):927-935.
6. Berlemann M. Does hurricane risk affect individual well-being? Empirical evidence on the indirect effects of natural disasters. *Ecological Economics.* 2016;124:99-113.
7. Seddighi H, Yousefzadeh S, López López M, Sajjadi H. Preparing children for climate-related disasters. *BMJ Paediatrics Open.* 2020;4(1):e000833.
8. Nguyen CV, Minh Pham N. The impact of natural disasters on children's education: Comparative evidence from Ethiopia, India, Peru, and Vietnam. *Review of Development Economics.* 2018;22(4):1561-1589.
9. Seddighi H, Salmani I, Javadi MH, Seddighi S. Child abuse in natural disasters and conflicts: a systematic review. *Trauma, Violence, & Abuse.* 2019:1524838019835973.
10. Hadjipanayis A, Crawley FP, Stiris T, Neubauer D, Michaud P-A. Child trafficking in Europe: what is the paediatrician's role? *European journal of pediatrics.* 2018;177(9):1419-1423.
11. Jones A. Malnutrition, poverty, and climate change are also human rights issues in child labor. *Health and human rights.* 2018;20(2):249.
12. Kumala Dewi LPR, Dartanto T. Natural disasters and girls vulnerability: is child marriage a coping strategy of economic shocks in Indonesia? *Vulnerable children and youth studies.* 2019;14(1):24-35.
13. Kakar S. Child/Forced/Servile Marriages⇄ Human Trafficking. *The Palgrave International Handbook of Human Trafficking.* 2020:503-519.
14. Sodhi MS. Natural disasters, the economy and population vulnerability as a vicious cycle with exogenous hazards. *Journal of Operations Management.* 2016;45:101-113.
15. Raccanello D, Gobbo C, Corona L, De Bona G, Hall R, Burro R. Long-term intergenerational transmission of memories of the Vajont disaster. *Psychological Trauma: Theory, Research, Practice, and Policy.* 2019.
16. Daoud A, Halleröd B, Guha-Sapir D. What is the association between absolute child poverty, poor governance, and natural disasters? A global comparison of some of the realities of climate change. *PLoS one.* 2016;11(4).
17. Midtbust LGH, Dyregrov A, Djup HW. Communicating with children and adolescents about the risk of natural disasters. *European journal of psychotraumatology.* 2018;9(sup2):1429771.
18. McNeill IM, Ronan KR. Children in disasters: the role of household preparedness. *Natural hazards.* 2017;89(3):1239-1254.
19. Muzenda-Mudavanhu C. A review of children's participation in disaster risk reduction. *Jàmbá: Journal of Disaster Risk Studies.* 2016;8(1).

Confidential: For Review Only

20. Zhu T-T, Zhang Y-J. An investigation of disaster education in elementary and secondary schools: evidence from China. *Natural hazards.* 2017;89(3):1009-1029.
21. Muñoz VA, Carby B, Abella EC, et al. Success, innovation and challenge: School safety and disaster education in South America and the Caribbean. *International Journal of Disaster Risk Reduction.* 2019:101395.
22. Moher D, Shamseer L, Clarke M, et al. Preferred reporting items for systematic review and meta-analysis protocols (PRISMA-P) 2015 statement. *Systematic Reviews.* 2015;4(1):1.
23. Higgins J, Wells G. Cochrane handbook for systematic reviews of interventions. 2011.
24. Higgins JP, Altman DG, Gøtzsche PC, et al. The Cochrane Collaboration's tool for assessing risk of bias in randomised trials. *Bmj.* 2011;343:d5928.
25. Institute JB. JBI critical appraisal checklist for case reports. 2017.
26. Hannes K. Supplementary guidance for inclusion of qualitative research in Cochrane systematic reviews of interventions. 2011.
27. Popay J, Roberts H, Sowden A, et al. Guidance on the conduct of narrative synthesis in systematic reviews. *A product from the ESRC methods programme Version.* 2006;1:b92.

Confidential: For Review Only

PRISMA-P 2015 Checklist

This checklist has been adapted for use with protocol submissions to *Systematic Reviews* from Table 3 in Moher D et al**:** Preferred reporting items for systematic review and meta-analysis protocols (PRISMA-P) 2015 statement. *Systematic Reviews* 2015 **4**:1

Section/topic	#	Checklist item	Information reported: Yes	Information reported: No	Line number(s)
**ADMINISTRATIVE INFORMATION**					
**Title:** **School-based education programs for preparing children for natural disasters: A Systematic Review Protocol**					
Identification	1a	Identify the report as a protocol of a systematic review	☒	☐	
Update	1b	If the protocol is for an update of a previous systematic review, identify as such	☐	☒	
**Registration**	2	If registered, provide the name of the registry (e.g., PROSPERO) and registration number in the Abstract	☒	☐	
**Authors**					
Contact	3a	Provide name, institutional affiliation, and e-mail address of all protocol authors; provide physical mailing address of corresponding author	☒	☐	
Contributions	3b	Describe contributions of protocol authors and identify the guarantor of the review	☒	☐	
**Amendments**	4	If the protocol represents an amendment of a previously completed or published protocol, identify as such and list changes; otherwise, state plan for documenting important protocol amendments	☐	☒	
**Support**					
Sources	5a	Indicate sources of financial or other support for the review	☒	☐	
Sponsor	5b	Provide name for the review funder and/or sponsor	☒	☐	
Role of sponsor/funder	5c	Describe roles of funder(s), sponsor(s), and/or institution(s), if any, in developing the protocol	☒	☐	
**INTRODUCTION**					
**Rationale**	6	Describe the rationale for the review in the context of what is already known	☒	☐	
**Objectives**	7	Provide an explicit statement of the question(s) the review will address with reference to participants, interventions, comparators, and outcomes (PICO)	☒	☐	

BioMed Central The Open Access Publisher

Confidential: For Review Only

Section/topic	#	Checklist item	Information reported		Line number(s)
			Yes	No	
**METHODS**					
**Eligibility criteria**	8	Specify the study characteristics (e.g., PICO, study design, setting, time frame) and report characteristics (e.g., years considered, language, publication status) to be used as criteria for eligibility for the review	☒	☐	
**Information sources**	9	Describe all intended information sources (e.g., electronic databases, contact with study authors, trial registers, or other grey literature sources) with planned dates of coverage	☒	☐	
**Search strategy**	10	Present draft of search strategy to be used for at least one electronic database, including planned limits, such that it could be repeated	☒	☐	
STUDY RECORDS					
Data management	11a	Describe the mechanism(s) that will be used to manage records and data throughout the review	☒	☐	
Selection process	11b	State the process that will be used for selecting studies (e.g., two independent reviewers) through each phase of the review (i.e., screening, eligibility, and inclusion in meta-analysis)	☒	☐	
Data collection process	11c	Describe planned method of extracting data from reports (e.g., piloting forms, done independently, in duplicate), any processes for obtaining and confirming data from investigators	☒	☐	
**Data items**	12	List and define all variables for which data will be sought (e.g., PICO items, funding sources), any pre-planned data assumptions and simplifications	☒	☐	
**Outcomes and prioritization**	13	List and define all outcomes for which data will be sought, including prioritization of main and additional outcomes, with rationale	☒	☐	
**Risk of bias in individual studies**	14	Describe anticipated methods for assessing risk of bias of individual studies, including whether this will be done at the outcome or study level, or both; state how this information will be used in data synthesis	☒	☐	
DATA					
**Synthesis**	15a	Describe criteria under which study data will be quantitatively synthesized	☒	☐	
	15b	If data are appropriate for quantitative synthesis, describe planned summary measures, methods of handling data, and methods of combining data from studies, including any planned exploration of consistency (e.g., I^2, Kendall's tau)	☐	☒	
	15c	Describe any proposed additional analyses (e.g., sensitivity or subgroup analyses, meta-regression)	☐	☒	
	15d	If quantitative synthesis is not appropriate, describe the type of summary planned	☒	☐	

Confidential: For Review Only

BioMed Central
The Open Access Publisher

3

Section/topic	#	Checklist item	Information reported		Line number(s)
			Yes	No	
**Meta-bias(es)**	16	Specify any planned assessment of meta-bias(es) (e.g., publication bias across studies, selective reporting within studies)	☐	☒	
**Confidence in cumulative evidence**	17	Describe how the strength of the body of evidence will be assessed (e.g., GRADE)	☒	☐	

Confidential: For Review Only

BioMed Central The Open Access Publisher

Additional file: Draft of the search strategy utilized in each selected database.

<table>
<tr><th colspan="2">Search Strategy</th></tr>
<tr><td>Population</td><td>child OR adolescent OR teen OR student OR boy OR girl OR kid OR pediatric</td></tr>
<tr><td colspan="2">AND</td></tr>
<tr><td rowspan="3">Interest</td><td>floods OR hurricanes OR tornadoes OR volcanoes OR earthquakes OR tsunamis OR storms OR hazards OR risks OR fire OR bushfire OR landslide OR sandstorm OR drought OR snowstorm OR avalanche OR thunderstorms OR COVID-19 OR Influenza OR Zika OR malaria OR Ebola OR epidemic</td></tr>
<tr><td>AND</td></tr>
<tr><td>preparedness OR education OR readiness OR drill</td></tr>
<tr><td colspan="2">AND</td></tr>
<tr><td>Context</td><td>school OR class</td></tr>
</table>

Confidential: For Review Only

Search Strategy – PubMed (May 5, 2020)

Search:

#1 (((child[Title/Abstract] OR adolescent[Title/Abstract] OR teen[Title/Abstract] OR student[Title/Abstract] OR boy[Title/Abstract] OR girl[Title/Abstract] OR kid[Title/Abstract] OR pediatric[Title/Abstract])

AND

#2 (floods[Title/Abstract] OR hurricanes[Title/Abstract] OR tornadoes[Title/Abstract] OR volcanoes[Title/Abstract] OR earthquakes[Title/Abstract] OR tsunamis[Title/Abstract] OR storms[Title/Abstract] OR hazards[Title/Abstract] OR risks[Title/Abstract] OR fire[Title/Abstract] OR bushfire[Title/Abstract] OR landslide[Title/Abstract] OR sandstorm[Title/Abstract] OR drought[Title/Abstract] OR snowstorm[Title/Abstract] OR avalanche[Title/Abstract] OR thunderstorms[Title/Abstract] OR COVID-19[Title/Abstract] OR Influenza[Title/Abstract] OR Zika[Title/Abstract] OR malaria[Title/Abstract] OR Ebola[Title/Abstract] OR epidemic[Title/Abstract]))

AND

#3 (preparedness[Title/Abstract] OR education[Title/Abstract] OR readiness[Title/Abstract] OR drill[Title/Abstract]))

AND

#4 (school[Title/Abstract] OR class[Title/Abs act]) (((((((("child"[Title/Abstract] OR "adolescent"[Title/Abstract]) OR "teen"[Title/Abstract]) OR "student"[Title/Abstract]) OR "boy"[Title/Abstract]) OR "girl"[Title/Abstract]) OR "kid"[Title/Abstract]) OR "pediatric"[Title/Abstract])

Confidential: For Review Only

Seacrch strategy in PUBMED

5/5/2020 Advanced Search Results - PubMed

Search	Actions	Details	Query	Results	Time
#1	•••		Search: **(((child[Title/Abstract] OR adolescent[Title/Abstract] OR teen[Title/Abstract] OR student[Title/Abstract] OR boy[Title/Abstract] OR girl[Title/Abstract] OR kid[Title/Abstract] OR pediatric[Title/Abstract]) AND (floods[Title/Abstract] OR hurricanes[Title/Abstract] OR tornadoes[Title/Abstract] OR volcanoes[Title/Abstract] OR earthquakes[Title/Abstract] OR tsunamis[Title/Abstract] OR storms[Title/Abstract] OR hazards[Title/Abstract] OR risks[Title/Abstract] OR fire[Title/Abstract] OR bushfire[Title/Abstract] OR landslide[Title/Abstract] OR sandstorm[Title/Abstract] OR drought[Title/Abstract] OR snowstorm[Title/Abstract] OR avalanche[Title/Abstract] OR thunderstorms[Title/Abstract] OR COVID-19[Title/Abstract] OR Influenza[Title/Abstract] OR Zika[Title/Abstract] OR malaria[Title/Abstract] OR Ebola[Title/Abstract] OR epidemic)[Title/Abstract])) AND (preparedness[Title/Abstract] OR education[Title/Abstract] OR readiness[Title/Abstract] OR drill[Title/Abstract])) AND (school[Title/Abstract] OR class[Title/Abstract])**<br>((((((((("child"[Title/Abstract] OR "adolescent"[Title/Abstract]) OR "teen"[Title/Abstract]) OR "student"[Title/Abstract]) OR "boy"[Title/Abstract]) OR "girl"[Title/Abstract]) OR "kid"[Title/Abstract]) OR "pediatric"[Title/Abstract]) AND (((((((((((((((((((((("floods"[Title/Abstract] OR "hurricanes"[Title/Abstract]) OR "tornadoes"[Title/Abstract]) OR "volcanoes"[Title/Abstract]) OR "earthquakes"[Title/Abstract]) OR "tsunamis"[Title/Abstract]) OR "storms"[Title/Abstract]) OR "hazards"[Title/Abstract]) OR "risks"[Title/Abstract]) OR "fire"[Title/Abstract]) OR "bushfire"[Title/Abstract]) OR "landslide"[Title/Abstract]) OR "sandstorm"[Title/Abstract]) OR "drought"[Title/Abstract]) OR "snowstorm"[Title/Abstract]) OR "avalanche"[Title/Abstract]) OR "thunderstorms"[Title/Abstract]) OR "COVID-19"[Title/Abstract]) OR "Influenza"[Title/Abstract]) OR "Zika"[Title/Abstract]) OR "malaria"[Title/Abstract]) OR "Ebola"[Title/Abstract]) OR (((((((("epidemic s"[All Fields] OR "epidemical"[All Fields]) OR "epidemically"[All Fields]) OR "epidemicity"[All Fields]) OR "epidemics"[MeSH Terms]) OR "epidemics"[All Fields]) OR "epidemic"[All Fields]) OR "epidemiology"[MeSH Subheading]) OR "epidemiology"[All Fields])))) AND ((("preparedness"[Title/Abstract] OR "education"[Title/Abstract]) OR "readiness"[Title/Abstract]) OR "drill"[Title/Abstract])) AND ("school"[Title/Abstract] OR "class"[Title/Abstract])<br>**Translations**<br>**epidemic:** "epidemic's"[All Fields] OR "epidemical"[All Fields] OR "epidemically"[All Fields] OR "epidemicity"[All Fields] OR "epidemics"[MeSH Terms] OR "epidemics"[All Fields] OR "epidemic"[All Fields] OR "epidemiology"[Subheading] OR "epidemiology"[All Fields]	3,913	14:44:40

Showing 1 to 1 of 1 entries

BMJ Paediatrics Open

Students' Preparedness for Disasters in Schools: A Systematic Review Protocol

Journal:	*BMJ Paediatrics Open*
Manuscript ID	bmjpo-2020-000913.R1
Article Type:	Protocol
Date Submitted by the Author:	14-Nov-2020
Complete List of Authors:	Seddighi, Hamed; University of Social Welfare and Rehabilitation Science<br>Sajjadi, Homeira; University of Social Welfare and Rehabilitation Science<br>Yousefzadeh, Sepideh; RUG, Campus Friesland<br>Lopez, Monica; RUG<br>Vameghi, Meroe; University of Social Welfare and Rehabilitation Science<br>Rafiey, Hassan; University of Social Welfare and Rehabilitation Science<br>Khankeh, Hamid Reza; University of Social Welfare and Rehabilitation Science<br>Garzon Fonseca, Magdalena; University of Groningen,
Keywords:	Adolescent Health

SCHOLARONE™
Manuscripts

I, the Submitting Author has the right to grant and does grant on behalf of all authors of the Work (as defined in the below author licence), an exclusive licence and/or a non-exclusive licence for contributions from authors who are: i) UK Crown employees; ii) where BMJ has agreed a CC-BY licence shall apply, and/or iii) in accordance with the terms applicable for US Federal Government officers or employees acting as part of their official duties; on a worldwide, perpetual, irrevocable, royalty-free basis to BMJ Publishing Group Ltd ("BMJ") its licensees and where the relevant Journal is co-owned by BMJ to the co-owners of the Journal, to publish the Work in this journal and any other BMJ products and to exploit all rights, as set out in our licence.

The Submitting Author accepts and understands that any supply made under these terms is made by BMJ to the Submitting Author unless you are acting as an employee on behalf of your employer or a postgraduate student of an affiliated institution which is paying any applicable article publishing charge ("APC") for Open Access articles. Where the Submitting Author wishes to make the Work available on an Open Access basis (and intends to pay the relevant APC), the terms of reuse of such Open Access shall be governed by a Creative Commons licence – details of these licences and which Creative Commons licence will apply to this Work are set out in our licence referred to above.

Other than as permitted in any relevant BMJ Author's Self Archiving Policies, I confirm this Work has not been accepted for publication elsewhere, is not being considered for publication elsewhere and does not duplicate material already published. I confirm all authors consent to publication of this Work and authorise the granting of this licence.

Confidential: For Review Only

Students' Preparedness for Disasters in Schools: A Systematic Review Protocol

Hamed Seddighi, Homeira Sajjadi, Sepideh Yousefzadeh, Mónica López López, Meroe Vameghi, Hassan Rafiey, Hamidreza Khankeh, Magdalena Garzon Fonseca

Hamed Seddighi: Student Research Committee, University of Social Welfare and Rehabilitation Sciences, Tehran, Iran,Hseddighi@gmail.com

Homeira Sajjadi: Social Determinants of Health Research Center, University of Social Welfare and Rehabilitation Sciences, Tehran, Iran, Safaneh_s@yahoo.com

Sepideh Yousefzadeh: Population Research Centre, Faculty of Spatial Sciences, University of Groningen, Groningen, the Netherlands, s.yousefzadeh@rug.nl

Mónica López López: Faculty of Behavioural and Social Sciences, University of Groningen, Groningen, the Netherlands, m.lopez.lopez@rug.nl

Meroe Vameghi: Social Welfare Management Research Center, University of Social Welfare and Rehabilitation Sciences, Tehran, Iran, m_vameghi@yahoo.com

Hassan Rafiey: Social Welfare Management Research Center, University of Social Welfare and Rehabilitation Sciences, Tehran, Iran, hassan441015@gmail.com

Hamidreza Khankeh: Health in Emergency and Disaster Research Center, University of Social Welfare and Rehabilitation Sciences, Tehran, Iran, hamid.khankeh@ki.se

Magdalena Garzon Fonseca (Corresponding author): Department of Child & Family Welfare, Graduate School of Behavioral Sciences, University of Groningen, m.garzon.fonseca@rug.nl

Keywords: Disasters, Natural hazards, Climate change, Children, Preparedness

Word count: 2200

Confidential: For Review Only

Abstract

Introduction

Children are one of the most vulnerable groups in disasters. Improving students' knowledge and skills to prepare for disasters can play a major role in children's health. School as a place to teach children can make a significant contribution to provide the necessary skills. This study aims to identify the effects, strengths and weaknesses of interventions in schools to prepare children for disasters.

Methods and analysis

We use Preferred Reporting Items for Systematic Review and Meta-Analysis (PRISMA) guidelines to develop a protocol for this systematic review. The included studies will report on the results of interventions targeting “schoolchildren” defined as individuals between 4 and under 18 years old studying in schools. Different electronic databases will be used for a comprehensive literature search, including MEDLINE, Web of Science, CINAHL, PsycINFO, Cochrane Register of Controlled Trials (CENTRAL) and EMBASE to identify the records that match the mentioned inclusion criteria published till December 2020. The main search terms are "disaster", "preparedness", "children", and "school". Four types of data will be extracted from the qualified studies including study characteristics (study design, year of publication, and geographical region where the study was conducted), participant characteristics (sample size, age, and gender), intervention characteristics (aim of intervention, intervention facilitators and barriers), and intervention outcomes. The quality appraisal of the selected papers will be conducted using Cochrane Collaboration’s Risk of Bias for quantitative studies and Critical Appraisal Skills Program (CASP) checklist for qualitative studies. We use a narrative synthesis for this systematic review. The narrative synthesis refers to an approach to systematic reviews which focuses mostly on applying words and texts to summarize and explain findings.

Ethics and dissemination

This paper is a part of a Ph.D. thesis of Hamed Seddighi at University of Social welfare and Rehabilitation Sciences with ethics code IR.USWR.REC.1399.008 approved by the Ethics Committee of the above-mentioned university.

PROSPERO registration number

Registration number: CRD42020146536

Confidential: For Review Only

What is already known on this topic?

- In previous studies were indicated that preparing children could be useful and effective (e.g., evacuation drills in Chile, preparing students for hurricane in Cuba, and tsunami preparedness in Japan). Innovative solutions using digital tools for preparing children for climate-related disasters are effective.
- Preparing marginalized school students for disasters is vital. Intersection of childhood and racial and ethnic social class, disability, gender and residence inequalities increases vulnerability and therefore increases disasters' risk for children.

What this study hopes to add?

- This study provides a comprehensive summary of studies investigating School-based education programs for preparing children for disasters.
- Provide useful information for policy makers, governments, researchers, and humanitarian organizations about state of art of programs for preparing children for disasters around the world.
- Highlight closing the gaps for preparing children for disasters such as earthquake, flood, and pandemics.

Confidential: For Review Only

1. Introduction

In recent decades, more disasters have occurred[1]. Disasters are one of the major threats to children's health[2]. According to the origin, disasters triggered by natural hazards and technological hazards. Natural hazards are categorized as geophysical (earthquakes, landslides, tsunamis and volcanic activity), hydrological (avalanches and floods), climatological (extreme temperatures, drought and wildfires), meteorological (cyclones and storms/wave surges) or biological (disease epidemics and insect/animal plagues)[3]. Technological hazards are such as conflicts, famine, displaced populations, industrial accidents[3]. The World Health Organization defined "All-hazard approach" as a concept acknowledging that, while hazards vary in source (natural, technological, societal), they often challenge health systems in similar ways. Thus, risk reduction, emergency preparedness, response actions and community recovery activities are usually implemented along the same model, regardless of the cause"[4].Children are one of the most vulnerable groups during and after disasters[5]. disasters can seriously damage children's physical and mental health[6]. It also harms children indirectly, so that its outcomes may last a lifetime[7,8]. In addition, disasters destroy children's educational infrastructure and disrupt learning in children [9]. Moreover, following disasters, violence against children increases in sexual, physical, psychological and neglect forms[10]. Child trafficking has been reported as one of the impacts of disasters in the affected areas[11]. Children who have lost their parents or caregivers and the children whose parents cannot afford to live will have to work after disasters[11,12]. Early marriage is another case reported in the affected areas, especially for girls[13,14]. Each of the above-mentioned cases also affects social, economic, and political structures[15]. These consequences will last for generations[16]. Although timely response and good governance have a significant effect in preventing such consequences, children's readiness can be one of the main solutions to reduce post-disaster losses[17,18].

In previous decades, most managers and decision-makers would think that children are not able to take care of themselves and they need help from adults[19]. Therefore, most interventions for disaster preparedness were defined for adults. But now researchers have found that children can respond appropriately to an accident and stay healthy[20]. The United Nations has recognized raising the children's awareness as one of the main pillars of children's readiness[20].

School as a place where almost all children gather to enjoy the opportunity to learn, can be the best place to prepare them for disasters[21]. The required knowledge and skill can be provided to the child in various ways in schools, one of which is practical training and skills development[22]. Although various studies have been conducted on the interventions in schools to prepare the children for disasters, no systematic review has been yet conducted to identify methods and challenges.

1.1. Aim of Review

The main purpose of this study is to identify the methods, outcomes, facilitators and barriers of educational programs in schools to prepare children for disasters. In addition, the differences of processes and outcomes in terms of type of disasters and social inequalities including geographical location, age, gender, disability, ethnic, and religion belief, are investigated. However, as the fundamental aim of the school-based programs is to prepare children in order to reduce trauma in those exposed. It is not possible for the proposed narrative synthesis to address the effectiveness of these programs in reducing trauma among exposed children

Confidential: For Review Only

2. Method

We use Preferred Reporting Items for Systematic Review and Meta-Analysis (PRISMA) guidelines to develop the protocol for this systematic review (Supplementary Material 1) [23] registered in PROSPERO International Prospective Register of Systematic Reviews (registration number: CRD42020146536). In addition, Cochrane guidance is applied in the development of a systematic review protocol to specify the research questions using PICO-elements (Population, Interventions, Comparators, and Outcomes). Studies will be assessed against clearly defined criteria to determine their inclusion or exclusion in the review. Finally, the findings of the included studies will be assessed and reported[24].

2.1. Eligibility

2.1.1. Inclusion Criteria

a) Type of participants:

The included studies will report on the results of interventions targeting "schoolchildren" defined as individuals under 18 years old studying in schools.

b) Context/Setting:

The age of compulsory school entry is various in the world but it is normally 4 to 7 years old. For this review we will consider the children aged 4 to 18 in public or private schools.

c) Type of Interventions:

The included studies will report on the interventions delivered in schools with a focus on disasters' preparedness education. Various interventions with different duration, timing, and modality will be acceptable. Acceptable interventions are those delivered in full or in part in the schools. Such interventions were delivered by teachers or other people playing a disaster preparedness role. Schools are chosen as the primary setting because many programs for children are delivered in schools (i.e. shakeout drills).

d) Type of studies:

The studies included in this review will be as the following: qualitative studies (those evaluating the process of school-based educational interventions for preparedness against disasters), observational studies (including case-control, cohort and cross-sectional studies) and clinical trials (including non-randomized and randomized controlled trials) examining the effect of disaster preparedness education interventions on school students.

e) Types of publication:

The study will include empirical research works published in peer-reviewed journals or conference proceedings that are accompanied with full-length peer-reviewed papers.

f) Outcome measures:

The included studies will report on the interventions the primary outcome of which is an appropriate focus on the children's preparedness (knowledge, attitude, and behavior) for disasters.

The primary goal of all included interventions is to improve children preparedness for disasters. For this reason, included studies shall report on at least one educational instrument applied to measure children's preparedness.

2.1.2. Exclusion Criteria

a) Type of participants:

The study will exclude individuals under 4 and above 18 years old.

Confidential: For Review Only

b) Context/setting:

Studies will be excluded if they report interventions delivered in settings other than the schools.

c) Type of interventions:

Studies will be excluded if (i) they do not include a disasters' preparedness intervention for improving skills and changing knowledge, attitude, and behavior, (ii) they report on the interventions delivered without a school-based component (i.e. disasters' preparedness education for children via television.)

d) Type of studies:

Studies will be excluded if they are not original intervention studies, they are published in journals that are not peer-reviewed. They will be excluded if they do not report on a disasters' preparedness.

2.2. Search Strategy

We will use electronic databases for comprehensive literature search including MEDLINE, Web of Science, CINAHL, PsycINFO, Cochrane Register of Controlled Trials (CENTRAL) and EMBASE to identify the records meeting the mentioned inclusion criteria and published till April 2020. Different keywords for the systematic search were identified during the initial literature search. The main search terms are "disaster", "preparedness", "children", and "school". The combinations of keywords related to population, interest, and context and the draft of MEDLINE search strategy is presented in appendix 1. Search terms will be combined with the appropriate Boolean operators and the search will be based on titles, abstracts, and keywords.

2.3. Selection Processes

Two reviewers will participate in the selection process. An Endnote desktop will be used to store references and subsequently identify and remove duplicates. For finding eligible studies, abstracts and full texts will be reviewed. Two reviewers will then separately scan abstracts and full texts of currently eligible studies against the eligibility requirements of the research, taking into consideration the type of intervention, sample population and the recorded outcomes. The two reviewers must come to an agreement through discussion and if they fail to reach a compromise, a third reviewer may have a resolution.

2.4. Data Extraction

Two reviewers shall individually perform data retrieval, and agreement will be achieved through conversation. The following data will be extracted from the qualified studies:

- Study characteristics: study design, year of publication, and geographical location of study conduct
- Participant characteristics: sample size, age (e.g., mean with standard deviation, range), and gender
- Intervention characteristics: aim of intervention, intervention facilitators and barriers,
- Intervention outcome

2.5. Quality Appraisal

The quality appraisal of the selected papers will be conducted separately by two reviewers using Cochrane Collaboration's Risk of Bias (CCRB)[25,26]. CCRB will be utilized for measuring risk of bias for selected articles with randomized design. Bias is surveyed in five dimensions, including selection bias, performance bias, detection bias, attrition bias, and reporting bias.

For assessing risk of bias in qualitative studies, we will use the Critical Appraisal Skills Program (CASP) checklist recommended by the Cochrane Collaboration for qualitative literature[27]. This tool has 10 questions (9 questions addressing quality and 1 addressing "value").

Confidential: For Review Only

2.6. Data Synthesis

Due to the heterogeneity and variation of the studies to be reviewed, especially wide range of disasters, we will not use a statistical aggregation of the data. Instead, a narrative synthesis will be used for this systematic review. In the narrative data synthesis, we will use from "all hazard approach" that was defined in the introduction. The narrative synthesis refers to an approach to systematic reviews that focuses mostly on use of words and texts to summarize and explain findings[28]. It is usually considered as the "second best" approach for synthesis in systematic reviews. In fact, this approach is a significant method to interpret findings extensively used in policy and practice. This approach will discuss on the effects of interventions and factors shaping the implementation of interventions. According to Popay et al. (2006), in order to conduct the narrative synthesis four steps shall be followed [28]:

- Developing a theory of how the intervention works, why and for whom,
- Developing a preliminary synthesis of findings of included studies,
- Exploring relationships in the data,
- Assessing the robustness of the synthesis.

the first step will contribute to the interpretation of the study findings and to assess how widely the findings may be applicable. The second step is developing a preliminary synthesis. In this step the aim is to identify and list facilitators and barriers to implementation of interventions on children's preparedness in schools for disasters. Exploring relationships in the data will be the third step. This step aims to explain the facilitators and/or barriers to successful implementation across the included studies and to understand how and why the interventions have an effect. In the final step, the robustness of the synthesis product will be assessed. The aim of this step is to provide an assessment of the strength of the evidence in order to draw conclusions about the facilitators and/or barriers to implementation identified in the synthesis.

for developing a preliminary synthesis, three methods will be used including textual descriptions, grouping and clusters, and tabulation.

In textual descriptions, a descriptive paragraph on the included studies will be presented. In grouping method, we aim to cluster findings to aid the process of analyzing and finding patterns. Studies will be categorized according to the type of interventions, contexts, and outcomes. In addition, tabulation will be used to summarize some findings.

3. Conclusions

In the planned systematic review, we aim to identify methods, outcomes, facilitators, and barriers of school-based education programs for preparing children for disasters. Such a review can raise decision-makers' knowledge of disaster preparedness for children. Recognizing best practices will be an opportunity for countries researching for interventions. Researchers can also find solutions to remove the barriers to preparedness of children for disasters.

Confidential: For Review Only

Supplementary Materials
PRISMA checklist
Search strategy

Contributors
HS is the guarantor. HS drafted the manuscript. HSJ, SY, HR, MV, HK and MGF provided edits for manuscript improvement. MV developed the search strategy. HSJ, SY and MLL contributed to the revision of the report. All authors reviewed, contributed to and approved the final version of the manuscript. All authors have read and agreed to the published version of the manuscript.

Funding and Ethics
This paper is a part of a Ph.D. thesis of Hamed Seddighi at University of Social welfare and Rehabilitation Sciences with ethics code IR.USWR.REC.1399.008 approved by the Ethics Committee of the mentioned university.

Competing interests: Not applicable.

Patient and public involvement: Not applicable.

Patient consent for publication: Not applicable.

Confidential: For Review Only

References

1. Joshi N, Roberts R, Tryggvason A. Are fatality potentials of natural disasters increasing? Examining how natural and human factors affect societal vulnerability. Paper presented at: Geophysical Research Abstracts2019.
2. Seddighi H, Sajjadi H, Salmani I. Child-Friendly Humanitarian Logistics in Natural Disasters: A Letter to the Editor. *Iranian Red Crescent Medical Journal.* 2019;21(8).
3. Guha-Sapir D, Vos F, Below R, Ponserre S. Annual disaster statistical review 2010. *Centre for Research on the Epidemiology of Disasters.* 2011.
4. World Health O. *Health emergency and disaster risk management framework.* Geneva: World Health Organization; 2019.
5. Benevolenza MA, DeRigne L. The impact of climate change and natural disasters on vulnerable populations: A systematic review of literature. *Journal of Human Behavior in the Social Environment.* 2019;29(2):266-281.
6. Subedi S, Bartels S, Davison C. Emotional and physical child abuse in the context of natural disasters: a focus on Haiti. *Disaster medicine and public health preparedness.* 2019;13(5-6):927-935.
7. Berlemann M. Does hurricane risk affect individual well-being? Empirical evidence on the indirect effects of natural disasters. *Ecological Economics.* 2016;124:99-113.
8. Seddighi H, Yousefzadeh S, López López M, Sajjadi H. Preparing children for climate-related disasters. *BMJ Paediatrics Open.* 2020;4(1):e000833.
9. Nguyen CV, Minh Pham N. The impact of natural disasters on children's education: Comparative evidence from Ethiopia, India, Peru, and Vietnam. *Review of Development Economics.* 2018;22(4):1561-1589.
10. Seddighi H, Salmani I, Javadi MH, Seddighi S. Child abuse in natural disasters and conflicts: a systematic review. *Trauma, Violence, & Abuse.* 2019:1524838019835973.
11. Hadjipanayis A, Crawley FP, Stiris T, Neubauer D, Michaud P-A. Child trafficking in Europe: what is the paediatrician's role? *European journal of pediatrics.* 2018;177(9):1419-1423.
12. Jones A. Malnutrition, poverty, and climate change are also human rights issues in child labor. *Health and human rights.* 2018;20(2):249.
13. Kumala Dewi LPR, Dartanto T. Natural disasters and girls vulnerability: is child marriage a coping strategy of economic shocks in Indonesia? *Vulnerable children and youth studies.* 2019;14(1):24-35.
14. Kakar S. Child/Forced/Servile Marriages⇄ Human Trafficking. *The Palgrave International Handbook of Human Trafficking.* 2020:503-519.
15. Sodhi MS. Natural disasters, the economy and population vulnerability as a vicious cycle with exogenous hazards. *Journal of Operations Management.* 2016;45:101-113.
16. Raccanello D, Gobbo C, Corona L, De Bona G, Hall R, Burro R. Long-term intergenerational transmission of memories of the Vajont disaster. *Psychological Trauma: Theory, Research, Practice, and Policy.* 2019.
17. Daoud A, Halleröd B, Guha-Sapir D. What is the association between absolute child poverty, poor governance, and natural disasters? A global comparison of some of the realities of climate change. *PLoS one.* 2016;11(4).
18. Midtbust LGH, Dyregrov A, Djup HW. Communicating with children and adolescents about the risk of natural disasters. *European journal of psychotraumatology.* 2018;9(sup2):1429771.
19. McNeill IM, Ronan KR. Children in disasters: the role of household preparedness. *Natural hazards.* 2017;89(3):1239-1254.

Confidential: For Review Only

20. Muzenda-Mudavanhu C. A review of children's participation in disaster risk reduction. *Jàmbá: Journal of Disaster Risk Studies.* 2016;8(1).
21. Zhu T-T, Zhang Y-J. An investigation of disaster education in elementary and secondary schools: evidence from China. *Natural hazards.* 2017;89(3):1009-1029.
22. Muñoz VA, Carby B, Abella EC, et al. Success, innovation and challenge: School safety and disaster education in South America and the Caribbean. *International Journal of Disaster Risk Reduction.* 2019:101395.
23. Moher D, Shamseer L, Clarke M, et al. Preferred reporting items for systematic review and meta-analysis protocols (PRISMA-P) 2015 statement. *Systematic Reviews.* 2015;4(1):1.
24. Higgins J, Wells G. Cochrane handbook for systematic reviews of interventions. 2011.
25. Higgins JP, Altman DG, Gøtzsche PC, et al. The Cochrane Collaboration's tool for assessing risk of bias in randomised trials. *Bmj.* 2011;343:d5928.
26. Institute JB. JBI critical appraisal checklist for case reports. 2017.
27. Hannes K. Supplementary guidance for inclusion of qualitative research in Cochrane systematic reviews of interventions. 2011.
28. Popay J, Roberts H, Sowden A, et al. Guidance on the conduct of narrative synthesis in systematic reviews. *A product from the ESRC methods programme Version.* 2006;1:b92.

Confidential: For Review Only

PRISMA-P 2015 Checklist

This checklist has been adapted for use with protocol submissions to *Systematic Reviews* from Table 3 in Moher D et al: Preferred reporting items for systematic review and meta-analysis protocols (PRISMA-P) 2015 statement. *Systematic Reviews* 2015 **4**:1

Section/topic	#	Checklist item	Information reported: Yes	Information reported: No	Section where each item is addressed in the protocol
**ADMINISTRATIVE INFORMATION**					
**Title**: **Students' Preparedness for Disasters in Schools: A Systematic Review Protocol**					
Identification	1a	Identify the report as a protocol of a systematic review	☒	☐	Title page
**Registration**	2	If registered, provide the name of the registry (e.g., PROSPERO) and registration number in the Abstract	☒	☐	Title page
**Authors**					
Contact	3a	Provide name, institutional affiliation, and e-mail address of all protocol authors; provide physical mailing address of corresponding author	☒	☐	Title page
Contributions	3b	Describe contributions of protocol authors and identify the guarantor of the review	☒	☐	Contributors
**Amendments**	4	If the protocol represents an amendment of a previously completed or published protocol, identify as such and list changes; otherwise, state plan for documenting important protocol amendments	☐	☒	n/a
**Support**					
Sources	5a	Indicate sources of financial or other support for the review	☒	☐	Funding
Sponsor	5b	Provide name for the review funder and/or sponsor	☒	☐	Funding
Role of sponsor/funder	5c	Describe roles of funder(s), sponsor(s), and/or institution(s), if any, in developing the protocol	☒	☐	Funding
**INTRODUCTION**					
**Rationale**	6	Describe the rationale for the review in the context of what is already known	☒	☐	What is already known on this topic?
**Objectives**	7	Provide an explicit statement of the question(s) the review will address with reference to participants, interventions, comparators, and outcomes (PICO)	☒	☐	Introduction
**METHODS**					

Confidential: For Review Only

Section/topic	#	Checklist item	Information reported Yes	No	Section where each item is addressed in the protocol
**Eligibility criteria**	8	Specify the study characteristics (e.g., PICO, study design, setting, time frame) and report characteristics (e.g., years considered, language, publication status) to be used as criteria for eligibility for the review	☒	☐	Eligibility
**Information sources**	9	Describe all intended information sources (e.g., electronic databases, contact with study authors, trial registers, or other grey literature sources) with planned dates of coverage	☒	☐	Search Strategy
**Search strategy**	10	Present draft of search strategy to be used for at least one electronic database, including planned limits, such that it could be repeated	☒	☐	Supplementary File 2
STUDY RECORDS					
Data management	11a	Describe the mechanism(s) that will be used to manage records and data throughout the review	☒	☐	Data Extraction
Selection process	11b	State the process that will be used for selecting studies (e.g., two independent reviewers) through each phase of the review (i.e., screening, eligibility, and inclusion in meta-analysis)	☒	☐	Selection Processes
Data collection process	11c	Describe planned method of extracting data from reports (e.g., piloting forms, done independently, in duplicate), any processes for obtaining and confirming data from investigators	☒	☐	Data Extraction
**Data items**	12	List and define all variables for which data will be sought (e.g., PICO items, funding sources), any pre-planned data assumptions and simplifications	☒	☐	Data Extraction
**Outcomes and prioritization**	13	List and define all outcomes for which data will be sought, including prioritization of main and additional outcomes, with rationale	☒	☐	Inclusion Criteria
**Risk of bias in individual studies**	14	Describe anticipated methods for assessing risk of bias of individual studies, including whether this will be done at the outcome or study level, or both; state how this information will be used in data synthesis	☒	☐	Quality Appraisal
DATA					
**Synthesis**	15a	Describe criteria under which study data will be quantitatively synthesized	☒	☐	Data synthesis
	15d	If quantitative synthesis is not appropriate, describe the type of summary planned	☒	☐	Data synthesis
**Meta-bias(es)**	16	Specify any planned assessment of meta-bias(es) (e.g., publication bias across studies, selective reporting within studies)	☐	☒	n/a
**Confidence in cumulative evidence**	17	Describe how the strength of the body of evidence will be assessed (e.g., GRADE)	☒	☐	Data synthesis

Confidential: For Review Only

Additional file: Draft of the search strategy utilized in each selected database.

Search Strategy	
**Population**	child *OR* adolescent *OR* teen *OR* student *OR* boy *OR* girl *OR* kid *OR* pediatric
AND	
**Interest**	floods *OR* hurricanes *OR* tornadoes *OR* volcanoes *OR* earthquakes *OR* tsunamis *OR* storms *OR* hazards *OR* risks *OR* fire *OR* bushfire *OR* landslide *OR* sandstorm *OR* drought *OR* snowstorm *OR* avalanche *OR* thunderstorms *OR* COVID-19 *OR* Influenza *OR* Zika *OR* malaria *OR* Ebola *OR* epidemic *OR* famine *OR* mass shooting *OR* industrial accident
	AND
	preparedness *OR* education *OR* readiness *OR* drill
AND	
**Context**	school *OR* class

Confidential: For Review Only

Search Strategy – PubMed (May 5, 2019)

Search:

#1 (((child[Title/Abstract] OR adolescent[Title/Abstract] OR teen[Title/Abstract] OR student[Title/Abstract] OR boy[Title/Abstract] OR girl[Title/Abstract] OR kid[Title/Abstract] OR pediatric[Title/Abstract])

AND

#2 (floods[Title/Abstract] OR hurricanes[Title/Abstract] OR tornadoes[Title/Abstract] OR volcanoes[Title/Abstract] OR earthquakes[Title/Abstract] OR tsunamis[Title/Abstract] OR storms[Title/Abstract] OR hazards[Title/Abstract] OR risks[Title/Abstract] OR fire[Title/Abstract] OR bushfire[Title/Abstract] OR landslide[Title/Abstract] OR sandstorm[Title/Abstract] OR drought[Title/Abstract] OR snowstorm[Title/Abstract] OR avalanche[Title/Abstract] OR thunderstorms[Title/Abstract] OR COVID-19[Title/Abstract] OR Influenza[Title/Abstract] OR Zika[Title/Abstract] OR malaria[Title/Abstract] OR Ebola[Title/Abstract] OR epidemic[Title/Abstract] OR conflicts[Title/Abstract] famine[Title/Abstract] OR mass shooting OR [Title/Abstract] OR industrial accidents [Title/Abstract]))

AND

#3 (preparedness[Title/Abstract] OR education[Title/Abstract] OR readiness[Title/Abstract] OR drill[Title/Abstract]))

AND

#4 (school[Title/Abstract] OR class[Title/Abs act]) (((((((("child"[Title/Abstract] OR "adolescent"[Title/Abstract]) OR "teen"[Title/Abstract]) OR "student"[Title/Abstract]) OR "boy"[Title/Abstract]) OR "girl"[Title/Abstract]) OR "kid"[Title/Abstract]) OR "pediatric"[Title/Abstract])

Confidential: For Review Only
